# Is high body fat estimated by body mass index and waist circumference a predictor of hypertension in adults? A population-based study

**DOI:** 10.1186/1475-2891-11-112

**Published:** 2012-12-17

**Authors:** Diego Augusto Santos Silva, Edio Luiz Petroski, Marco Aurelio Peres

**Affiliations:** 1 Post-graduation Program in Physical Education, Federal University of Santa Catarina, Florianópolis, Brazil; 2 Post-graduation Program in Public Health, Federal University of Santa Catarina, Florianópolis, Brazil

**Keywords:** Anthropometry, Risk factors, Obesity, Hypertension, Adults

## Abstract

**Background:**

The aim of this study was to assess the predictive capacity of body fat percentage (%BF) estimated by equations using body mass index (BMI) and waist circumference (WC) to identify hypertension and estimate measures of association between high %BF and hypertension in adults.

**Methods:**

This is a cross-sectional population-based study conducted with 1,720 adults (20–59 years) from Florianopolis, southern Brazil. The area under the ROC curve, sensitivity, specificity, predictive values, and likelihood ratios of cutoffs for %BF were calculated. The association between %BF and hypertension was analyzed using Poisson regression, estimating the unadjusted and adjusted prevalence ratios and 95% CI.

**Results:**

The %BF equations showed good discriminatory power for hypertension (area under the ROC curve > 0.50). Considering the entire sample, the cutoffs for %BF with better properties for screening hypertension were identified in the equation with BMI for men (%BF = 20.4) and with WC for women (%BF = 34.1). Adults with high %BF had a higher prevalence of hypertension.

**Conclusions:**

The use of simple anthropometric measurements allowed identifying the %BF, diagnosing obesity, and screening people at risk of hypertension in order to refer them for more careful diagnostic evaluation.

## Background

Obesity is a universal disease of increasing prevalence with alarming proportions worldwide, including in countries that recently had hunger and chronic malnutrition as main public health problems [1]. Obesity is the major risk factor for hypertension, being responsible for 20% to 30% of disease cases. In 2009, hypertension accounted for 7.5 million (12.8%) deaths worldwide [2].

Among the methods used to assess body fat, anthropometric indicators stand out and, because they are inexpensive and noninvasive and have good accuracy, are recommended by the World Health Organization for assessing the nutritional status and health risk of populations [3]. Body fat percentage (%BF) is usually assessed by anthropometry by measuring skinfolds due to their high correlation with body fat [4]. The correct measurement of skinfolds demands substantial time and requires a high degree of training and expertise of evaluators [5], which impair the use of these measures in multidisciplinary epidemiological surveys that collect large amounts of data about a given population [6].

Clinical studies have developed equations to assess %BF by means of anthropometric indicators of obesity, such as body mass index (BMI) and waist circumference (WC), which are more easily collected than skinfolds [7-9]. These equations show a strong association with body fat when estimated by more accurate methods, such as dual energy X-ray absorptiometry (DEXA) and hydrostatic weighing.

The % BF estimation allows for more precise analysis of body components than simply BMI and WC measures. Through % BF, lean body mass and fat mass can be estimated [10]. In this sense, if equations that estimate % BF have good predictive capacity for hypertension, they can, while analyzing body composition, identify people at risk for hypertension.

Physical education, sports, and nutrition professionals routinely assess body fat in populations. These professionals have been associated with health teams; for example, in 2008, the Brazilian government regulated these professions into primary health care teams [11]. The identification of discriminatory power and the screening features of %BF equations using indicators such as BMI and WC can be useful for professionals that take care of a large number of individuals. If these equations show good predictive and screening characteristics, health professionals will be able not only to use anthropometry as a tool for assessing obesity but also to identify people at risk for hypertension and refer them for monitoring and diagnostic tests. Moreover, the evaluation of the specific role of anthropometric indices on the development of hypertension may help to better understand the pathogenesis of arterial hypertension, and to provide more accurate means for prevention [12].

This study aims to analyze the discriminatory power and screening properties of %BF assessed by equations with BMI and WC in the identification of hypertension. In addition, the associations between high %BF and hypertension are assessed in a sample of adults in southern Brazil.

## Methods

### Population and sample

This cross-sectional population-based study originated from the health survey *EpiFloripa Adults 2009* conducted in Florianópolis, Santa Catarina, southern Brazil, between September 2009 and January 2010. Florianópolis is the capital of the Brazilian state of Santa Catarina and has close to 420,000 inhabitants; it is also the Brazilian capital with the best social and health indicators [13].

The sample size was calculated to estimate the prevalence of each health outcome investigated in the survey, given a reference population of 249,530 adults from 20 to 59 years of age, a confidence level of 95%, 50% prevalence for unknown health outcomes, an error sample of 3.5 percentage points, a design effect (deff) estimated at 2 due to cluster sampling, and percentage of losses estimated at 10%. Based on these parameters, a sample size of 1,720 individuals was obtained. However, considering the multiple objectives of the *EpiFloripa* study, this study interviewed 32 adults in each of the 63 census sectors, thus increasing the sample size to 2,016 individuals.

Sampling was conducted in two stages. In the first, the 420 urban census sectors of the city were stratified according to deciles of the family head income (R$ 192.80 to R$ 13.209.50 [Brazilian currency], US$ 1 = R$ 1.7 during the data collection period); 60 sectors were randomly selected (sampling fraction equal to seven), consisting of six sectors in each decile. In the second stage, the sampling units were the households. In this step, the number of permanent private households in each sector was updated by the study supervisors. The number of dwellings ranged from 61 to 810, giving a variation coefficient of occupied households between sectors of 55%. To decrease this, and considering the geographical proximity and income decile, it was decided to group some census sectors and divide others, resulting in 63 census sectors with a variation coefficient of 32%. Eighteen households from each of these geographical units were randomly selected.

### Eligibility, exclusion, and loss criteria

All adults aged 20 to 59 years living in selected households were eligible for the study. The exclusion criteria for carrying out anthropometric measurements and blood pressure measures were bedridden subjects, amputees, plastered and pregnant women, and those who had given birth in the six months preceding the interview. Individuals with neurological disorders that might interfere with their understanding the questions regarding the survey interview were also excluded. One resident who was not found in at least four visits, one in the weekend and another at night, was considered lost.

### Data collection

Data collection was carried among all adults living in selected households. To this end, 35 interviewers were selected. A personal digital assistant (PDA) was used to record and store data. All interviewers were intensively trained for the fieldwork; the survey pretest was conducted among 30 adults not sampled, and the pilot study was conducted among 100 individuals.

### Outcome

The dependent variable was hypertension (yes/no), which was defined as systolic pressure ≥ 140 mmHg and/or diastolic pressure ≥ 90 mmHg, and/or self-report of taking some antihypertensive medication, and/or diagnosis of hypertension by a doctor [14].

Blood pressure levels were measured twice, and the average measurements were used in the study. The resting time was approximately 30 minutes before the first measurement and approximately 15 minutes between measures. Blood pressure was measured in accordance with the recommendations of the Brazilian Guidelines on Hypertension [15]. For this purpose, blood pressure was measured on the right arm, which rested on a table at heart level with the palm facing upward. The individual remained sitting with his feet planted on the ground. At the beginning of the interview, the individuals were advised to refrain from smoking and drinking coffee, *chimarrão*, or black tea, and to empty their bladder. Electronic sphygmomanometers with a digital readout system (Techiline®, São Paulo, Brazil), previously and properly calibrated by the National Institute of Metrology, Standardization and Industrial Quality (Inmetro), were used to measure blood pressure levels.

### Anthropometric variables

Anthropometric measurements of body mass, height, and WC were evaluated according to standardized procedures [16], and the mean of two measurements was used in the study.

Body mass was measured with the digital scale model HCM 5110M (GA.MA Italy Professional®, Bologna, Italy), with a resolution of 100 grams and 150 kg capacity, which was calibrated before the survey. Height was measured using a stadiometer with a measuring tape at a resolution of 1 mm. The WC, taken without any clothing, was measured with an inextensible anthropometric tape (Sanny ®, São Bernardo do Campo, Brazil) with a resolution of 1 mm at the narrowest waist.

%BF was calculated based on equations with BMI [8] and WC [9]. The equation using BMI developed with a sample composed of white Americans and Africans showed a determination coefficient of R = 0.86 and a standard error of 4.98% when compared to the body composition analysis of the four-compartment model evaluated by means of DEXA (bone density) and hydrostatic weighing (water and body volume) [8]. The equation of Gallagher et al. [8] is as follows: *%BF = 64.5 - 848 x (1/BMI) + 0.079 x age - 16.4 x sex + 0.05 x sex x age + 39.0 x sex x (1/BMI)*. In the indication of sex, the values are 1 for men and 0 for women; age is given in complete years.

The equation with WC developed with healthy adults from Glasgow, Scotland, showed a determination coefficient of R = 77.8%, with %BF estimated by means of hydrostatic weighing, and a standard error of 4.10% for men, and a determination coefficient of R = 70.4% and a standard error of 4.70% for women [9]. The equation of Lean et al. [9] differs between men and women. *For men: %BF = (0.567 x WC) + (0.101 x age) - 31.8. For women: %BF = (0.439 x WC) + (0.221 x age) - 9.4*. WC must be in cm, and age in complete years.

BMI was calculated (weight/height ^2^) and ranked according to the literature [3] into obesity (BMI ≥ 30 kg/m^2^), overweight (BMI of 25.0 to 29.9 kg/m^2^) normal weight (BMI ≥ 18.5 and <25 kg/m^2^), and underweight (BMI <18.5). WC was assessed based on cutoff points in relation to risk of metabolic complications, and ranked into very high risk (men ≥ 102 cm, women ≥ 88 cm), high risk (men ≥ 94 cm, women ≥ 80 cm), and no risk (men <94 cm, women <80 cm) [3]. The inter-and intra-examiner technical error of measurement (TEM) was calculated according to the recommendations of Gore et al. [5]. The maximum inter-examiner TEM (1.86%) and intra-examiner TEM (1.18%) was found in the WC measure, which indicated an adequate level of interviewers for anthropometric measurements.

### Control variables

The control variables included demographic data, such as age, which was expressed in complete years and categorized as 20–39 and 40–59 years. The self-reported skin color was collected based on the categories proposed by the IBGE [17] and classified as white, light-skinned black, dark-skinned black, yellow, and indigenous. The socioeconomic variables were educational level assessed by the complete years of schooling and *per capita* family income in reais (R$, the Brazilian currency; US$ 1 = R$ 1.7 during the data collection period).

Smoking was assessed by the categories nonsmoker, former smoker, light smoker (less than 10 cigarettes daily), and moderate/heavy smoker (more than 20 cigarettes daily). The Alcohol Use Disorders Identification Test (AUDIT) was used to identify people with problematic alcohol use, using the cutoff point to classify subjects into no (score 0–7) and yes (score ≥ 8) [18]. Physical activity was assessed according to the leisure domain of the questionnaire used in the surveillance system of risk and protective factors for chronic diseases through a telephone survey (VIGITEL), considering as inactive those who practiced no physical exercise or practiced less than once a week in the three months preceding the interview [19]. Food habits were assessed based on the regular consumption of fruits and vegetables (≥ 5 days per week) according to the VIGITEL questionnaire [20].

### Statistical analysis

Descriptive statistics using the mean, standard deviation, and absolute and relative frequency was applied. To compare continuous variables between sexes, Student’s t-test for independent samples was used. To determine differences between sexes in the distribution of categorical variables, the chi-square test was used. The ROC curve was calculated to analyze the discriminatory power of %BF in the identification of hypertension and to find the best cutoffs to identify this association [21]. In this study, this cutoff point was the one with the best accuracy, i.e., with fewer false positives and false negatives. The larger the area under the ROC curve (AUC), the greater the discriminatory power of the %BF equation to identify hypertension. The confidence interval was calculated at 95% (95% CI), which determines whether or not the predictive capacity is due to chance, and the lower limit should not be less than 0.50 [22]. The differences between the AUC values of the different %BF equations were compared using the nonparametric test [23]. The sensitivity, specificity, positive predictive value (PPV), negative predictive value (NPV), positive likelihood ratio (LR +), and negative likelihood ratio (LR-) of all the best cutoffs of %BF were calculated to identify hypertension.

The association between hypertension and high %BF estimated by the cutoff points calculated in this study was analyzed by Poisson regression, estimating the crude and adjusted prevalence ratios and 95% CI. Three models of adjusted analysis were developed to determine the magnitude of association between excess fat and hypertension. Model 1 was adjusted by sociodemographic variables (age, skin color, educational level, and *per capita* family income). Model 2 was adjusted by sociodemographic variables and health behaviors (smoking, problematic alcohol use, practice of physical activities during leisure time, and regular consumption of fruits and vegetables). Model 3 was adjusted by sociodemographic variables, health behaviors, height, and BMI (for %BF equation with WC) or WC (for %BF equation with BMI). All analyses were stratified by sex and carried out considering the design effect and sampling weight. To calculate the screening properties of %BF equations, the MedCalc software version 12.1.4 was used. For association analyses, the Stata 9.0 software was used.

### Ethical aspects

The study was approved by the Ethics Committee on Human Research of the Federal University of Santa Catarina (No. 351/08). All the subjects who participated in the study signed an informed consent form.

## Results

The response rate of this study was 85.3% (*n* = 1720). The men had higher *per capita* family income, body mass, height, WC, and systolic and diastolic blood pressure values than the women (p <0.05). The men also had a higher prevalence of moderate/heavy smoking (13.1%), problematic alcohol use (29.7%), inadequate consumption of fruits and vegetables (86.6%), overweight (37.4%), and high %BF (43.1% for the equation with WC) compared to the women (8.0%, 9.6%, 76.9%, 26.4%, and 33.3%, respectively). On the other hand, the women had a higher prevalence of physical inactivity (58.6%) and abdominal obesity (20.4%) than the men (46.3% and 11.2%, respectively) (Table 1).

**Table 1:** Characteristics of the adult population

**Variables**	**Women**		**Men**	
	**n**	$\overset{―}{X}$**(CI 95%)**	**n**	$\overset{―}{X}$**(CI 95%)**
***Total***	959		761	
*Age (years)*	959	38.5 (37.8- 39.3)	761	37.4 (36.5- 38.2)
*Educational level*	958	11.6 (11.3- 11.9)	758	11.7 (11.4 - 12.0)
*Per capita family income* (R$)*†*	940	1.311.3 (1.219-3- 1.403.3)	745	1586.5 (1.420.0- 1.753.1)*
*Body mass (kg)*	925	65.3 (64.4- 66.2)	752	77.9 (76.9- 78.9)*
*Height (cm)*	928	160.4 (160.0- 160.9)	756	173.2 (172.7- 173.7)*
*Body mass index (kg/m*^*2*^*)*	923	25.4 (25.1- 25.7)	751	25.9 (25.6- 26.2)
*Waist circumference (cm)*	920	79.3 (78.4- 80.1)	751	88.4 (87.5- 89.2)*
*Fat percentage (equation with BM I)*	923	33.0 (32.5-33.4)*	751	20.9 (20.5-21.3)
*Fat percentage (equation with WCI)*	920	34.0 (33.5-34.5)*	751	22.1 (21.6-22.6)
*Systolic blood pressure (mmHg)*	928	127.5 (126.2- 128.6)	754	139.8 (138.4- 141.1)*
*Diastolic blood pressure ( (mmHg)*	928	81.9 (81.0- 82.8)	754	88.7 (87.6- 89.7)*
	**n**	**% (95% CI)**	**n**	**(95% CI)**
*Skin color*				
White	802	83.7 (79.8- 87.5)	642	84.1 (79.9 – 87.6)
Light-skinned black	73	8.0 (5.7- 10.2)	74	10.1 (6.8- 13.4)
Dark-skinned black	53	5.3 (3.0- 7.6)	34	4.5 (2.6- 6.6)
Yellow	15	1.7 (0.8 - 2.5)	02	0.3 (0.1 - 0.6)
Indigenous	12	1.3 (0.6 - 2.1)	08	1.0 (0.3 - 1.7)
*Age group*				
20-39 years	500	53.9 (49.6-58.1)	432	57.7 (52.3-63.0)
40-59 years	459	46.1 (41.9-50.3)	329	42.3 (37.0-47.7)
*Smoking*				
No smoker	548	57.4 (53.2- 61.5)	378	51.3 (46.1- 56.5)
Former smoker	238	25.1 (21.2- 28.9)	211	27.3 (22.3- 32.4)
Light smoker	92	9.5 (7.6- 11.4)	66	8.3 (6.1- 10.4)
Moderate / heavy smoker	77	8.0 (6.1- 9.9)	101	13.1 (10.2- 15.8)*
Problematic alcohol use				
No	870	90.4 (87.7- 93.0)	533	70.3 (66.1- 74.6)
Yes	89	9.6 (7.0- 12.3)	228	29.7 (25.3- 35.9)*
*Physical activity at leisure time*				
Active	400	41.4 (36.6- 46.1)	406	53.7 (48.1- 59.2)
Inactive	558	58.6 (53.8- 63.3)*	354	46.3 (40.7- 51.8)
*Food habit*				
Adequate	222	23.1 (19.8- 26.3)	101	13.4 (10.1- 16.8)
Inadequate	736	76.9 (73.7- 80.2)	660	86.6 (83.2- 89.9)*
*BMI*				
<18.5 kg/m^2^	26	2.8 (1.6- 4.0)	8	1.0 (0.2- 1.7)
18.5 – 24.9 kg/m^2^	493	54.1 (50.0- 58.2)	346	46.9 (42.6- 51.0)
25.0 – 29.9 kg/m^2^	249	26.4 (23.4- 29.3)	282	37.4 (34.1- 40.7)*
≥ 30.0 kg/m^2^	155	16.7 (13.7- 19.6)	115	14.7 (11.8- 17.4)
*WC (cm) ‡*				
No risk	536	58.6 (53.8- 63.4)	541	73.1 (69.2- 76.9)
High risk	188	21.0 (17.8- 24.1)	119	15.7 (12.9- 18.5)
Very high risk	196	20.4 (17.1- 23.6)*	91	11.2 (8.8- 13.6)
*Fat percentage (equation with BMI)*				
Normal	568	62.4 (58.2-66.5)	451	61.0 (56.7-65.4)
Increased	364	37.6 (33.4-41.7)	299	39.0 (34.6-43.3)
*Fat percentage (equation with WC)*				
Normal	610	66.7 (62.2-71.2)	421	56.9 (52.3-61.4)
Increased	309	33.3 (28.7-37.7)	329	43.1 (38.5-47.7)*
*Hypertension*				
No	642	69.5 (65.4- 73.6)	360	48.4 (44.2- 52.5)
Yes	286	30.5 (26.4- 34.6)	394	51.6 (47.5- 55.7)*

CI 95%: confidence interval of 95%; $\overset{―}{X}$**-** mean; *significant difference between sexes (p<0.05); †R$: Brazilian currency, 1US$ = 1,7R$ during the data collection period; BMI: Body mass index; WC: waist circumference.

**Figure 1:**
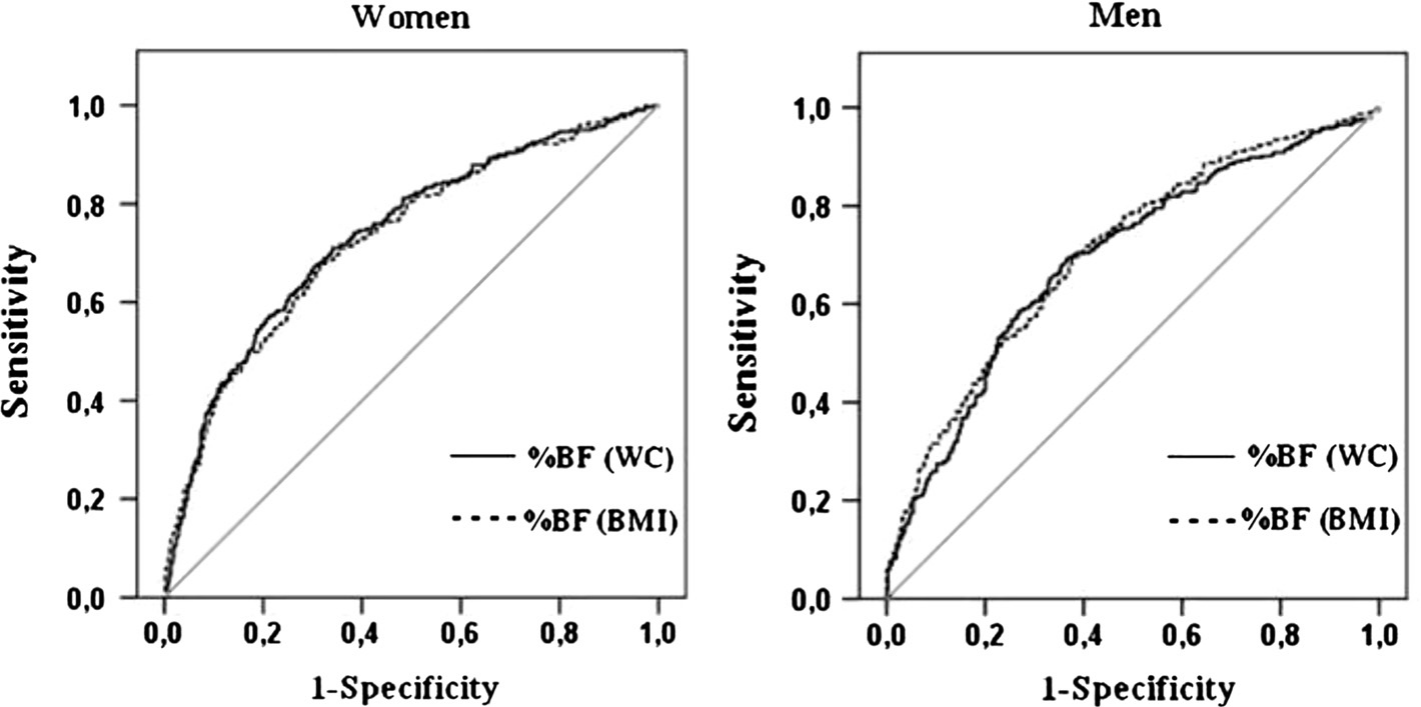
ROC curve analysis of body fat percentage (%BF) estimated by BMI and WC.

In both sexes (Table 2 and Figure 1), all %BF equations showed significant discriminatory power for hypertension (i.e., AUC > 0.5). The AUC did not differ between sexes or between %BF equations.

**Table 2:** Screening properties of %BF estimated by equation using BMI and WC to detect hypertension

	**AUC (95% CI)**	**Cutoff**	**Sensitivity(95% CI)**	**Specificity (95% CI)**	**PPV (95% CI)**	**NPV (95% CI)**	**LR+ (95% CI)**	**LR- (CI 95%)**
*Women*								
%BF (equation with BMI)	0.73 (0.70- 0.76)	34.0	68.1% (62.3- 73.4)	68.2% (64.4- 71.8)	49.1% (44.1- 54.2)	82.6% (79.0- 85.7)	2.1 (1.9- 2.4)	0.5 (0.4- 0.6)
%BF (equation with WC)	0.73 (0.70- 0.76)	34.1	71.0% (65.4- 76.2)	65.8% (61.9- 69.5)	48.2% (43.3- 53.1)	83.5% (79.9- 86.7)	2.1 (1.9- 2.3)	0.4 (0.4- 0.5)
*Men*								
%BF (equation with BMI)	0.70 (0.67- 0.73)	20.4	71.4% (66.6- 75.8)	59.8% (54.5- 65.0)	66.0% (61.2- 70.5)	65.7% (60.3- 70.9)	1.8 (1.6- 2.0)	0.5 (0.4- 0.6)
%BF (equation with WC)	0.69 (0.66- 0.73)	20.7	69.7% (64.8- 74.2)	63.4% (58.1- 68.4)	67.6% (62.8- 72.1)	65.6% (60.3- 70.6)	1.9 (1.7- 2.1)	0.5 (0.4- 0.6)

AUC: area under the curve, 95% CI: 95% confidence interval, PPV: positive predictive value, NPV: negative predictive value, LR+: positive likelihood ratio, LR-: negative likelihood ratio.

Note: The cutoff points units are: kg/m^2^, No significant difference between area under the curve by women and men, and by age (p>0.05).

Table 2 shows the screening properties for hypertension of the cutoffs of %BF estimated by the equation with BMI and WC. For women, the cutoff of %BF estimated by the equation with BMI with greater screening capacity (highest sensitivity values) for hypertension was 34.0%. This cutoff point showed high sensitivity (68.1%) and high NPV (82.6%). Additionally, this cutoff point showed LR+ of 2.1 and LR- of 0.5. In women, the cutoff of %BF estimated by the equation with WC with greater screening capacity for hypertension was 34.1%. This cutoff point showed sensitivity of 71.0% and NPV of 83.5%. LR+ and LR- were 2.1 and 0.4, respectively.

In men, the cutoff of %BF estimated by the equation with BMI with greater screening capacity for hypertension was 20.4%. This cutoff point had sensitivity of 71.4%, NPV of 65.7%, LR + of 1.8, and LR- of 0.5. For men, the cutoff of %BF estimated by the equation with WC with greater screening capacity for hypertension was 20.7%. This cutoff point showed sensitivity of 69.7% and NPV of 65.6%. LR+ and LR- were 1.9 and 0.5, respectively (Table 2).

**Figure 2:**
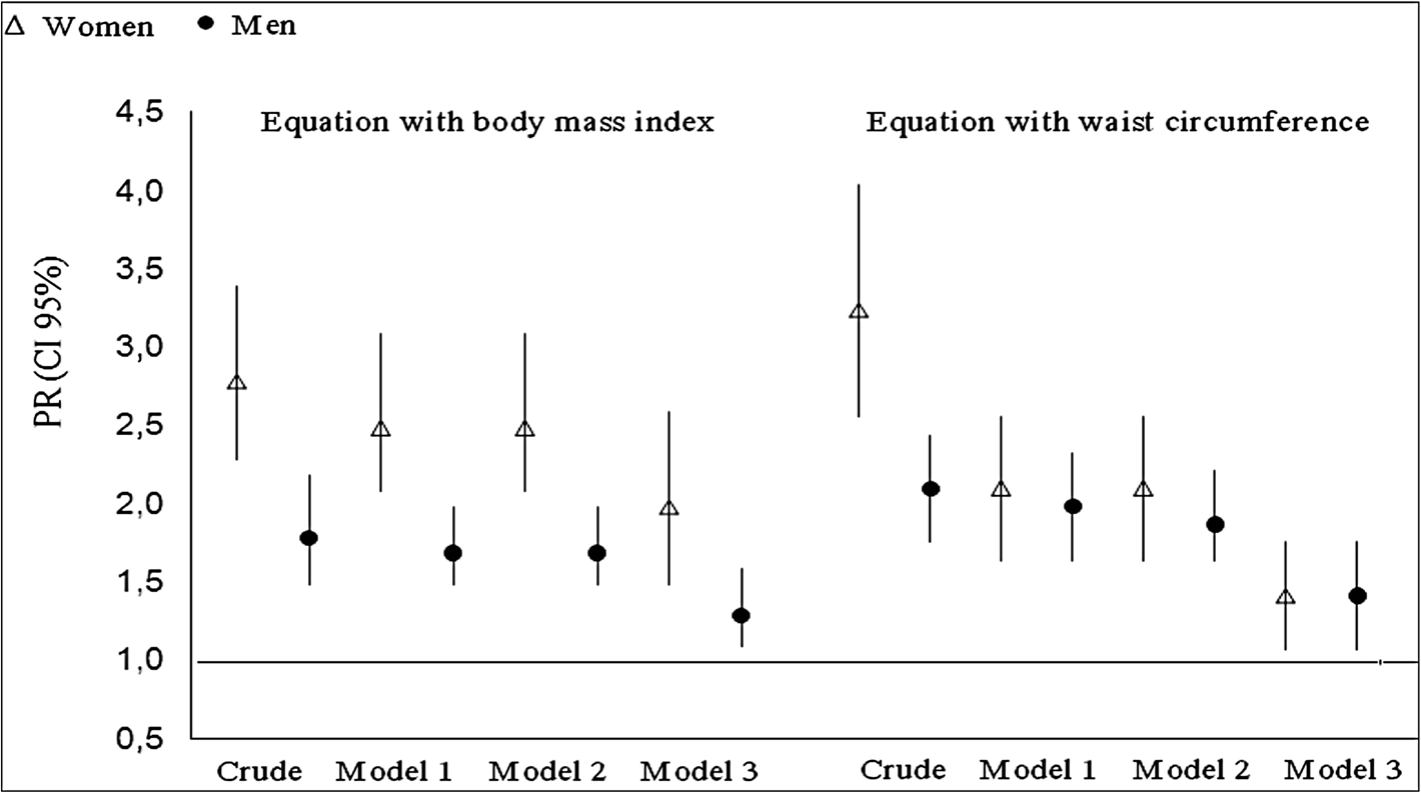
**Association between high %BF estimated by equations with BMI and WC and hypertension in adults.** PR: Prevalence ratios; CI95%: confidence intervals of 95%. Model 1: Analysis adjusted by sociodemographic variables (age, skin color, educational level and *per capita* family income). Model 2: Analysis adjusted by sociodemographic variables and health behaviors (smoking, problematic alcohol use, physical activity and consumption of fruits and vegetables). Model 3: Analysis adjusted by sociodemographic variables, health behaviors, height and waist circumference (for %BF estimated by equations with BMI) or body mass index (for %BF estimated by equations with WC).

Figures 2 show the measures of association between high %BF and hypertension. In all groups analyzed, adjusting the model by sociodemographic variables (model 1) and health behaviors (model 2) slightly modified the strength of association between high %BF and hypertension in both sexes compared to the unadjusted model. However, by adjusting the model simultaneously by sociodemographic variables, health behaviors, height, and WC or BMI (model 3), a reduction in this association was observed. The measures of association between exposure and outcome were higher for women than for men.

## Discussion

The predictive capacity of the %BF equations analyzed in this study was significant in identifying subjects with hypertension (i.e., AUC > 0.50). The literature [24,25] shows that a good screening test, in addition to having significant predictive capacity, should be inexpensive, easy to apply, and noninvasive, which are characteristics present in equations analyzed. However, another characteristic of a good screening test is high sensitivity so as to minimize the number of false negatives [24]. In this study, for women and men, the equations showed high sensitivity.

Screening is the search for any sign or symptom that might be indicative of a probable disease in asymptomatic subjects. In this study, screening is the identification of high %BF as indicative of hypertension. The purpose of screening is to refer people with any signs of the disease for better assessment, with monitoring and diagnosis from a qualified professional. Therefore, screening tests should be more "sensitive" and diagnostic tests more "specific" [25]. Thus, the %BF estimated by the equations analyzed and the cutoff points established in this study may be useful in screening hypertension in women and men. For women, the % BF equation with WC showed higher sensitivity values than the % BF equation with BMI. For men, the % BF equation with BMI showed higher sensitivity values than the % BF equation with WC.

One possible explanation for differences in sensitivity values between equations may be the origin of the population under study. The equation with WC used in this study was created from a sample composed of adults from Scotland, whose population is predominantly Caucasian [9]. The equation with BMI used in this study was developed with a sample of white Americans and African Americans [8]. The literature indicates that there are differences in fat distribution according to ethnicity. Asians, for example, have a higher adiposity degree for a given BMI compared to the white population [26]. The Brazilian population is very heterogeneous in terms of ethnicity, which can be verified by the distribution of the skin color variable.

The present study is consistent with other works in reporting that high body fat is associated with hypertension [27-29]. The crude measures of association for both equations and sexes indicate prevalence approximately twice as high of subjects with high %BF of having hypertension when compared to those with normal %BF. After adjusting by sociodemographic variables and health behaviors, this association was little changed, indicating that regardless of age, skin color, educational level, socioeconomic level, and lifestyle variables, excess fat is strongly associated with hypertension. This highlights why obesity is a major risk factor for hypertension, causing up to 30% of disease cases [2].

As in other studies that investigated the association between obesity and noncommunicable chronic diseases [28-30], this study found that by adjusting the analysis model by anthropometric indicators (BMI, WC, and height), the association between hypertension and high %BF decreased. This suggests that there is a high correlation between the anthropometric markers analyzed [29]. For example, an elevated BMI may be due to the accumulation of abdominal fat; high WC levels may be indicative of excess total body fat.

Through % BF, it is possible to estimate other body composition components [10]. Moreover, analyzing only the absolute WC and BMI values does not allow estimating lean body mass and fat mass. Thus, it is believed that the % BF estimation should also be considered in body fat and obesity evaluations at population level as widely performed with WC and BMI [3].

This study has some limitations: 1) the cross-sectional design does not allow establishing causal relationships between hypertension and high %BF so reverse causality cannot be ruled out; 2) the analysis of %BF conducted by means of anthropometric indicators of obesity is less accurate than other methods aimed at assessing body composition; and 3) chronic diseases, such as hypertension, are multifactorial, and in addition to anthropometric measurements, other factors, such as heredity, should be considered in future studies.

## Conclusions

Screening for hypertension using %BF estimated by equations with BMI and WC should be performed carefully, taking into account the properties of each measure. In practical terms, the screening of hypertension through %BF is more useful in women, regardless of age, if the equation with WC is used. In men, regardless of age, the screening is most useful if the %BF equation with BMI is used. Moreover, there is greater prevalence of hypertension in adults with high %BF values compared to those with lower %BF measurements.

The findings of this study show that while people with obesity are identified through % BF equations, there are individuals at risk of hypertension. This information may be relevant to health systems in countries of low and middle income that do not have enough financial and material resources for the accurate diagnosis of obesity and hypertension.

## Competing interests

The authors declare that they have no competing interests.

## Authors’ contributions

DASS conceived the study, supervised data collection and data analysis, and drafted the manuscript. ELP assisted in the statistical analysis, contributed to the interpretation of the results and drafted the manuscript. MAP is the day-to-day project manager, assisted in the statistical analysis and contributed to the interpretation of the results. All authors read and approved the final manuscript.
